# Hepatic HuR protects against the pathogenesis of non-alcoholic fatty liver disease by targeting PTEN

**DOI:** 10.1038/s41419-021-03514-0

**Published:** 2021-03-04

**Authors:** Mi Tian, Jingjing Wang, Shangming Liu, Xinyun Li, Jingyuan Li, Jianmin Yang, Cheng Zhang, Wencheng Zhang

**Affiliations:** 1 The Key Laboratory of Cardiovascular Remodeling and Function Research, Chinese Ministry of Education, Chinese National Health Commission and Chinese Academy of Medical Sciences, The State and Shandong Province Joint Key Laboratory of Translational Cardiovascular Medicine, Department of Cardiology, Qilu Hospital, Cheeloo College of Medicine, Shandong University, Jinan, China; 2 Cardiovascular Disease Research Center of Shandong First Medical University, Central Hospital Affiliated to Shandong First Medical University, Shandong, China; 3 Department of Physiology & Pathophysiology, School of Basic Medical Sciences, Shandong University, Shandong, China; 4 Department of Histology and Embryology, School of Basic Medical Sciences, Shandong University, Shandong, China

**Keywords:** Type 2 diabetes, Dyslipidaemias

## Abstract

The liver plays an important role in lipid and glucose metabolism. Here, we show the role of human antigen R (HuR), an RNA regulator protein, in hepatocyte steatosis and glucose metabolism. We investigated the level of HuR in the liver of mice fed a normal chow diet (NCD) and a high-fat diet (HFD). HuR was downregulated in the livers of HFD-fed mice. Liver-specific HuR knockout (HuR^LKO^) mice showed exacerbated HFD-induced hepatic steatosis along with enhanced glucose tolerance as compared with control mice. Mechanistically, HuR could bind to the adenylate uridylate-rich elements of phosphatase and tensin homolog deleted on the chromosome 10 (PTEN) mRNA 3′ untranslated region, resulting in the increased stability of *Pten* mRNA; genetic knockdown of HuR decreased the expression of PTEN. Finally, lentiviral overexpression of PTEN alleviated the development of hepatic steatosis in HuR^LKO^ mice in vivo. Overall, HuR regulates lipid and glucose metabolism by targeting PTEN.

## Introduction

Non-alcoholic fatty liver disease (NAFLD) is becoming the most common liver disease that leads to end-stage liver lesions with cardiovascular and metabolic comorbidity^1^. It is a pathological process that encompasses a spectrum of liver metabolic disorders starting with non-inflammatory liver steatosis, defined as >5% triglyceride (TG) accumulation in hepatocytes, which then leads to inflammation, fibrosis, and cirrhosis^2^.

Steatosis develops due to increased fatty acid (FA) uptake and de novo lipogenesis, which are associated with changes in hepatic glucose metabolism, including enhanced glycolysis and decreased gluconeogenesis^3,4^. Glucose and lipid metabolism are regulated by multiple mechanisms. The phosphatase and tensin homolog deleted on the chromosome 10 (PTEN)/phosphoinositide 3 kinase (PI3K)/AKT pathway has an important role because it acts downstream of the insulin receptor^5^. PTEN is a bispecific phosphoinositide and protein phosphatase that dephosphorylates phosphatidylinositol‑3,4,5‑trisphosphate (PIP3), terminating signaling downstream of phosphatidylinositol 3-kinase (PI3K), and decreasing AKT activity^6^. PTEN haploinsufficiency promotes insulin hypersensitivity^7^. Liver-specific PTEN deficiency promotes NAFLD and tumorigenesis while improving glucose tolerance^3,8^. PTEN is an important regulator of lipogenesis, glucose metabolism, and hepatocyte homeostasis in the liver. Thus, it is important to understand the regulation of PTEN expression and activity; however, the molecular mechanisms involved in PTEN expression have not been fully elucidated.

Human antigen R (HuR), also known as embryonic lethal abnormal vision-like 1 (ELAVL1), is a universally expressed member of the Hu/ELAV family of RNA-binding proteins^9^. As an RNA regulator, HuR selectively binds to adenylate uridylate-rich elements (AREs), which are usually found in the 3′ untranslated regions (UTR) of its targets via its RNA recognition motifs^10^. Global HuR-deficient mice show embryonic lethality due to extraembryonic placental defects^11^. Our previous study found that mice with adipose-specific HuR deletion were susceptible to obesity induced by a high-fat diet (HFD)^12^. In addition, HuR regulates cellular cholesterol homeostasis through modulating ATP-binding cassette transporter A1 (ABCA1) expression^13^. Moreover, HuR prevents HFD-induced NAFLD in mice by regulating lipid transport and ATP synthesis^14^. However, the specific role of HuR in hepatic steatosis related glucose metabolism has not been explicitly explored.

In this study, we investigated the role of HuR in the progression of HFD-induced hepatic steatosis and insulin resistance. Liver-specific HuR knockout (HuR^LKO^) mice were generated and treated with a HFD. We found that HuR deletion in the liver aggravated HFD-induced hepatic steatosis but alleviated HFD-induced insulin resistance by targeting PTEN.

## Results

### HuR expression was downregulated in hepatic steatosis

**Fig. 1:**
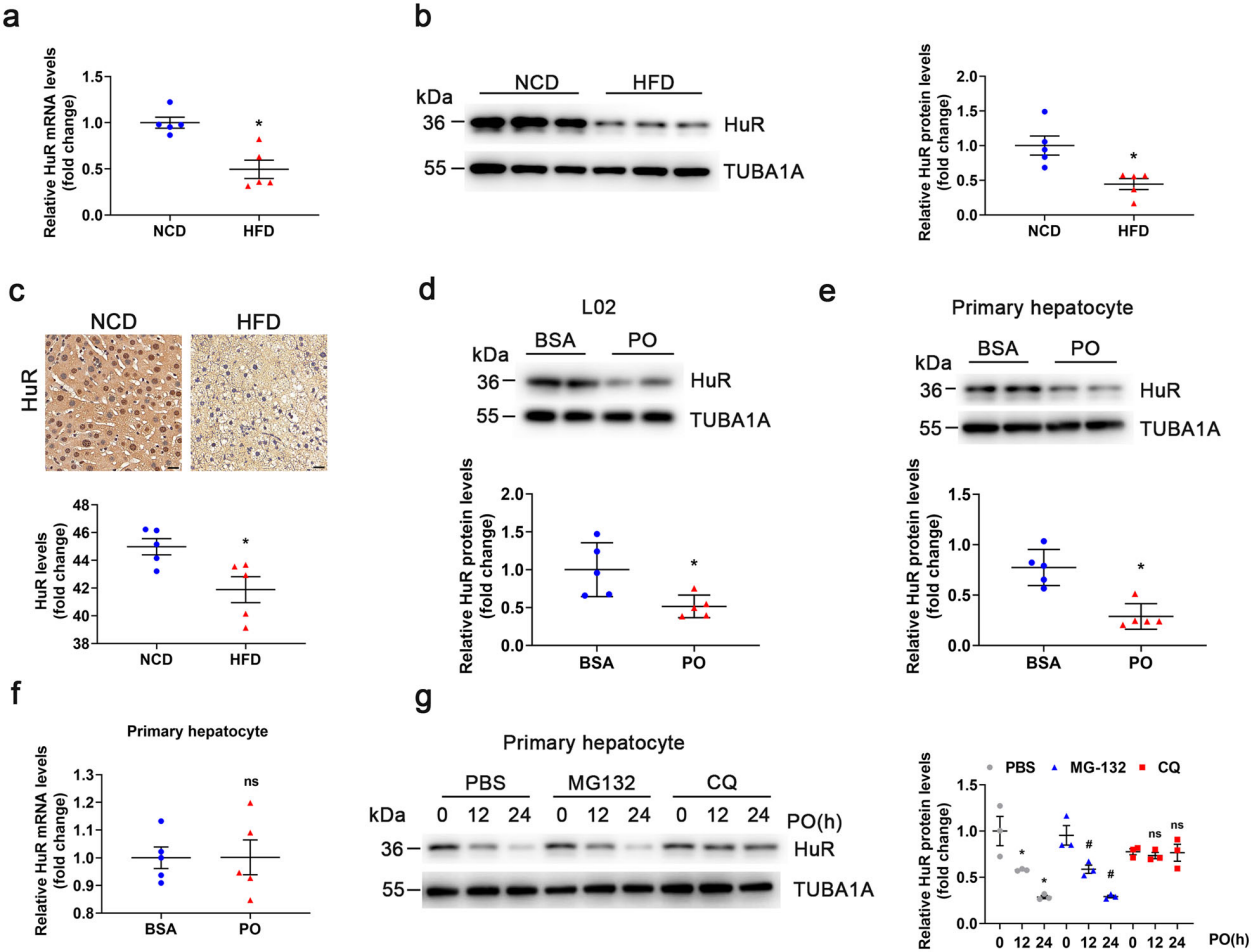
HuR expression was downregulated in hepatic steatosis. **a** qPCR analysis of HuR mRNA expression in livers of C57BL/6 J mice fed a NCD or HFD for 24 weeks (*n* = 5). *P < 0.05 vs NCD. **b** Western blot analysis of HuR protein levels in livers of C57BL/6J mice fed a NCD or HFD for 24 weeks (*n* = 5). *P < 0.05 vs NCD. **c** Immunohistochemical staining of HuR protein in livers from NCD- and HFD-fed mice (*n* = 5). *P < 0.05 vs NCD. Scale bar, 20 μm. Western blot analysis of HuR protein levels in L02 cells (**d**) and mouse primary hepatocytes (**e**) stimulated with BSA or PO (0.5 mM palmitic acid and 1.0 mM oleic acid) for 24 h (*n* = 5). *P < 0.05 vs BSA. **f** qPCR analysis of HuR mRNA levels in primary hepatocytes stimulated with BSA or PO for 24 h. **g** Primary hepatocytes were treated with PO and PBS, MG132, or CQ for 0, 12, or 24h. Western blot analysis of HuR is shown (*n* = 3). *P < 0.05 vs PO and PBS at 0 h. ^#^*P* < 0.05 vs PO and MG132 at 0 h.

To explore the function of HuR in hepatic steatosis, we evaluated its expression in the livers of C57BL/6J mice fed a HFD for 24 weeks. Quantitative polymerase chain reaction (qPCR), western blot analysis, and immunohistochemistry showed that HuR levels were significantly decreased in the livers of mice fed a HFD as compared with the controls (Fig. 1a–c). To detect HuR expression in the early stage of hepatic steatosis, C57BL/6J mice were fed a HFD for 2, 4, and 6 weeks. The levels of HuR mRNA and protein were significantly downregulated at the 6th week (Supplementary Fig. 1). Therefore, HuR may play a potential role in the development of NAFLD. Similarly, in L02 cells and primary hepatocytes stressed with palmitic acid and oleic acid (PO), HuR protein levels were also downregulated (Fig. 1d, e). However, HuR mRNA levels were not significantly regulated in primary hepatocytes stimulated with PO (Fig. 1f), which suggests that the downregulation of HuR protein might be a post-translational regulatory mechanism. To verify this hypothesis, primary hepatocytes were treated with PO, the proteasome inhibitor MG132, or the lysosome inhibitor chloroquine (CQ). Downregulation of HuR under PO stimulation was prevented by CQ treatment (Fig. 1g), suggesting that PO-induced HuR reduction via lysosomal degradation in hepatocytes.

### Liver-specific HuR deletion aggravated HFD-induced hepatic steatosis

To assess the role of HuR, particularly in the liver, liver-specific HuR-knockout (HuR^LKO^) mice were created (Supplementary Fig. 2a). The lack of HuR in the mouse liver tissue was confirmed via qPCR (Supplementary Fig. 2b). HuR protein expression was significantly decreased in the liver but not in the other tissues of HuR^LKO^ mice (Supplementary Fig. 2c), which was further confirmed by immunohistochemistry (Supplementary Fig. 2d).

**Fig. 2:**
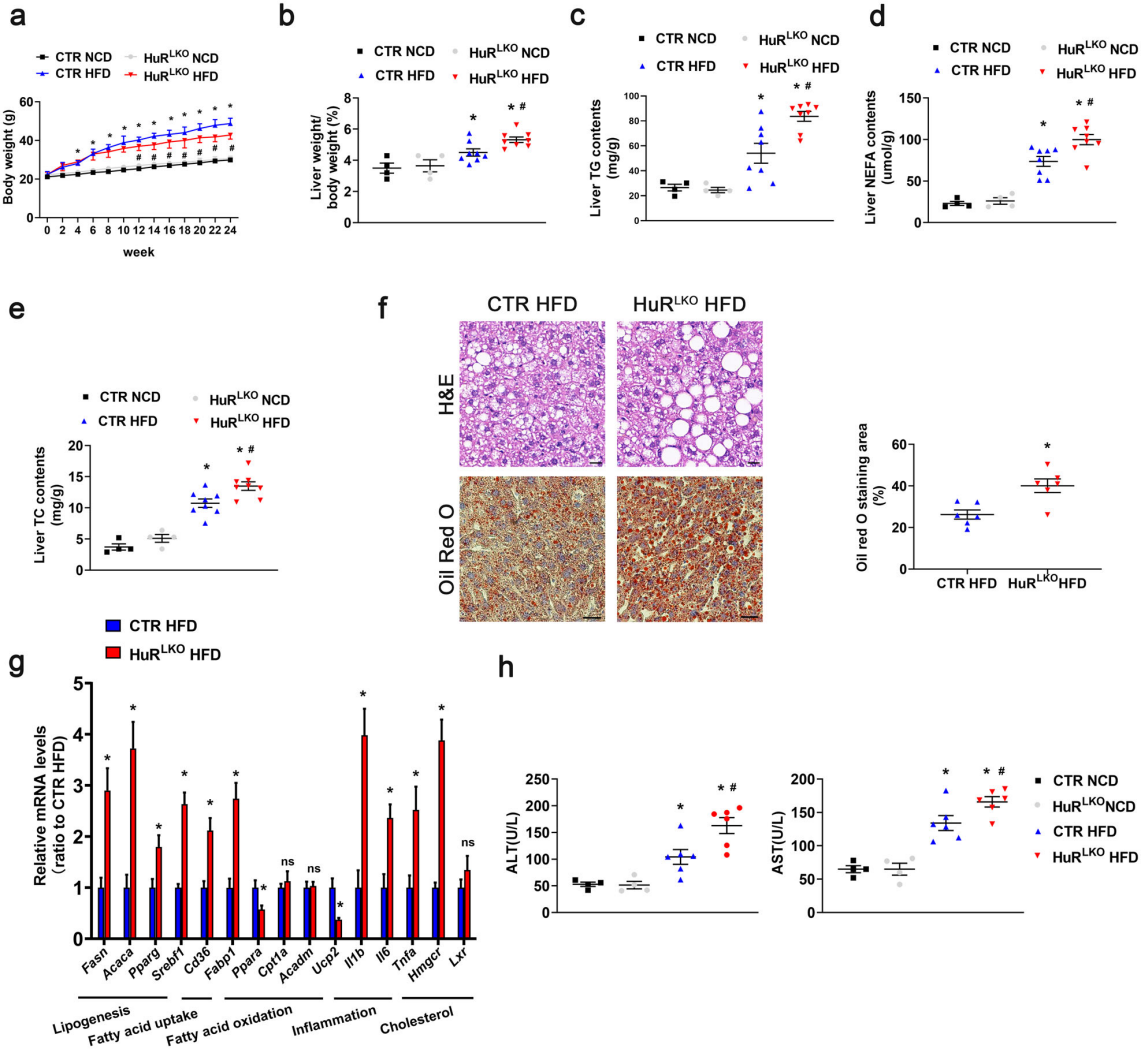
Liver-specific HuR deletion aggravated HFD-induced hepatic steatosis. Control and HuR^LKO^ mice were fed a NCD or HFD for 24 weeks. **a** Body weight of mice in different groups (*n* = 8). *P < 0.05 vs CTR NCD, ^#^*P* < 0.05 vs CTR HFD. **b** Liver weight to body weight ratio of mice in different groups (*n* = 8). *P < 0.05 vs CTR NCD, ^#^*P* < 0.05 vs CTR HFD. Triglycerides (TG) (**c**), nonesterified fatty acid (NEFA) (**d**), and cholesterol (TC) (**e**) levels in the liver of mice (*n* = 8). *P < 0.05 vs CTR NC, ^#^*P* < 0.05 vs CTR HFD. **f** H&E-stained (top) and Oil Red O-stained (bottom) liver sections from mice. Scale bar, 20 μm. Quantitative analysis (right) of the mean Oil-red O-stained area (*n* = 6). *P < 0.05 vs CTR HFD. **g** Relative mRNA levels of indicated molecules in the liver of mice (*n* = 5). *P < 0.05 vs CTR HFD. **h** Serum ALT and AST levels (*n* = 6). *P < 0.05 vs CTR NCD, ^#^*P* < 0.05 vs CTR HFD.

The 8-week-old control and HuR^LKO^ mice were fed a normal chow diet (NCD) or HFD for 24 weeks. HuR^LKO^ mice did not show overt abnormalities with NCD (Fig. 2a–f). However, under HFD, HuR^LKO^ mice gained less body weight, but a greater liver weight/body weight (LW/BW) ratio compared with the controls (Fig. 2a, b). Importantly, HuR^LKO^ mice showed exacerbated HFD-induced hepatic steatosis, as indicated by the lipid content [(TG, nonesterified fatty acids (NEFAs), and total cholesterol (TC)] (Fig. 2c–e). The greater lipid accumulation in HuR^LKO^ mice fed HFD was evident upon H&E and Oil Red O staining (Fig. 2f).

In addition, the mRNA levels of lipid metabolism-associated markers were measured. HuR deletion increased the mRNA levels of fatty acid uptake markers, such as *Cd36* and *Fabp1*, and lipogenesis markers, such as *Fasn*, *Acaca*, *Pparg*, and *Srebf1* (Fig. 2g). The levels of the key enzymes controlling liver fatty acid β-oxidation (*Ppara* and *Ucp2*) were reduced (Fig. 2g). Meanwhile, HuR deficiency increased the expression of the cholesterol metabolism molecule, *Hmgcr*, and inflammatory cytokines, such as interleukin 1 beta (*Il1β*), interleukin 6 (*Il6*), and tumor necrosis factor α (*Tnfa*; Fig. 2g). In addition, the serum alanine aminotransferase (ALT) and aspartate aminotransferase (AST) levels were increased in HuR^LKO^ mice compared to controls fed the HFD (Fig. 2h). These data suggest that HFD-induced lipid accumulation and inflammation were exaggerated after HuR deletion in the liver.

### Liver-specific HuR deletion alleviated HFD-impaired glucose tolerance

**Fig. 3:**
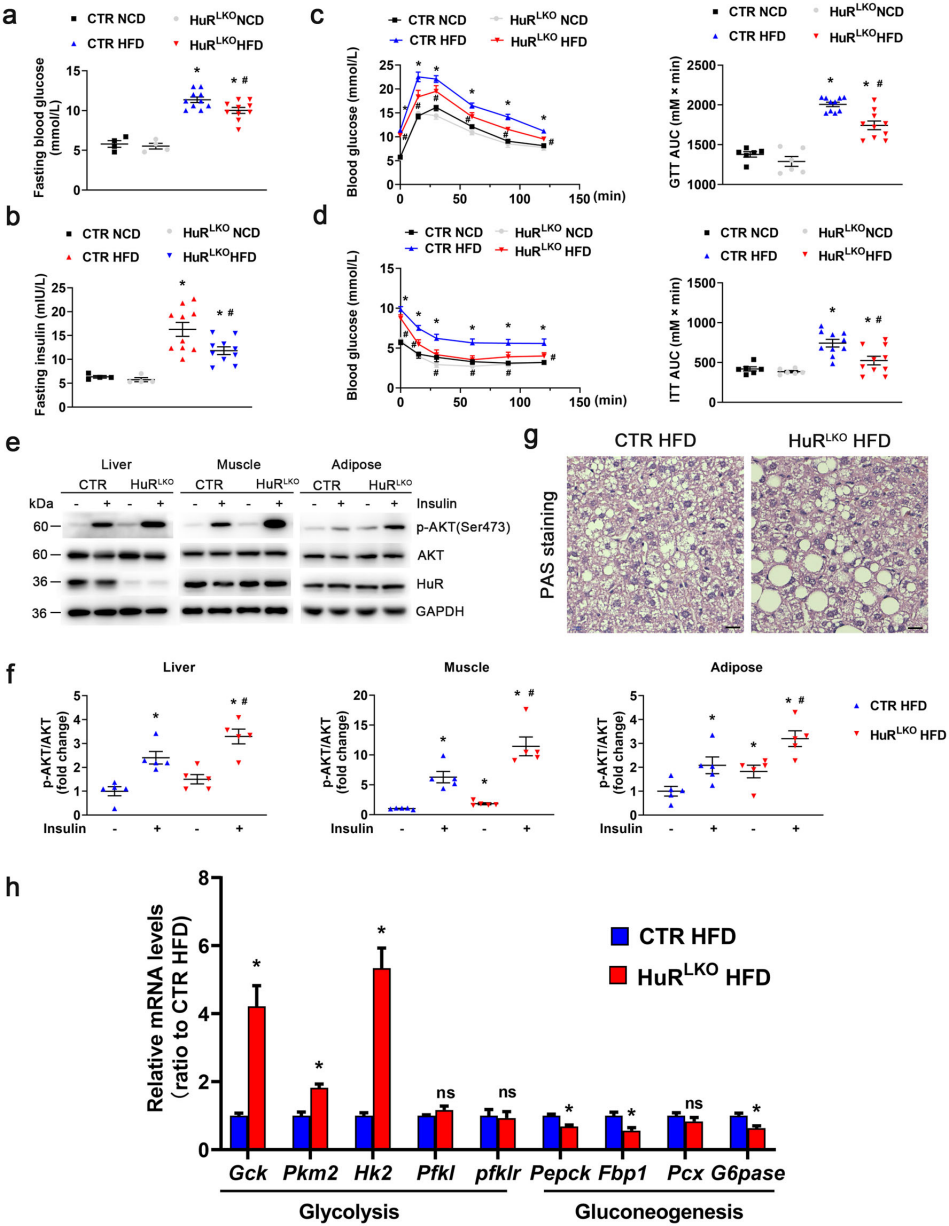
Liver-specific HuR deletion alleviated HFD-impaired glucose tolerance. Fasting glucose levels (**a**) and fasting insulin levels (**b**) of HuR^LKO^ and CTR mice in response to HFD (*n* = 10). *P < 0.05 *vs* CTR NCD, ^#^*P* < 0.05 vs CTR HFD. Glucose tolerance test (**c**) and insulin tolerance test (**d**) in HuR^LKO^ and CTR mice after NCD or HFD for 24 weeks. The corresponding areas under the curve (AUC) of blood glucose levels (right) (*n* = 8). *P < 0.05 vs CTR NCD, ^#^*P* < 0.05 vs CTR HFD. **e**, **f** Western blot analysis of AKT phosphorylation in tissues of control and HuR^LKO^ mice on HFD (*n* = 5). *P < 0.05 vs CTR HFD saline, ^#^*P* < 0.05 vs CTR HFD insulin. **g** Periodic acid-Schiff-stained liver sections from HuR^LKO^ and CTR mice after HFD feeding for 24 weeks. Scale bar, 20 μm. **h** Relative mRNA levels of genes involved in hepatic glycolysis and gluconeogenesis in HuR^LKO^ and CTR mice on HFD (*n* = 5). *P < 0.05 vs CTR HFD.

Hepatosteatosis is usually closely associated with impaired glucose tolerance. However, fasting glucose and insulin levels were significantly decreased in HuR^LKO^ mice compared with controls fed HFD (Fig. 3a, b). Glucose tolerance tests (GTT) and insulin tolerance tests (ITT) also indicated that HuR^LKO^ mice exhibited improved glucose tolerance and insulin resistance when fed HFD (Fig. 3c, d), which was confirmed by insulin-stimulated AKT Ser473 analysis in liver, skeletal muscle, and adipose tissue (Fig. 3e, f). Furthermore, HuR deletion increased the mRNA levels of glycolysis markers, including *Gck*, *Pkm2*, and *Hk2*, but decreased the expression of gluconeogenesis markers, such as *Pepck*, *Fbp1*, and *G6pase* (Fig. 3h). However, liver glycogen content was not affected by HuR knockout (Fig. 3g). Taken together, liver-specific HuR deletion alleviated the HFD-impaired glucose tolerance.

### HuR inhibited lipid accumulation in hepatocytes

**Fig. 4:**
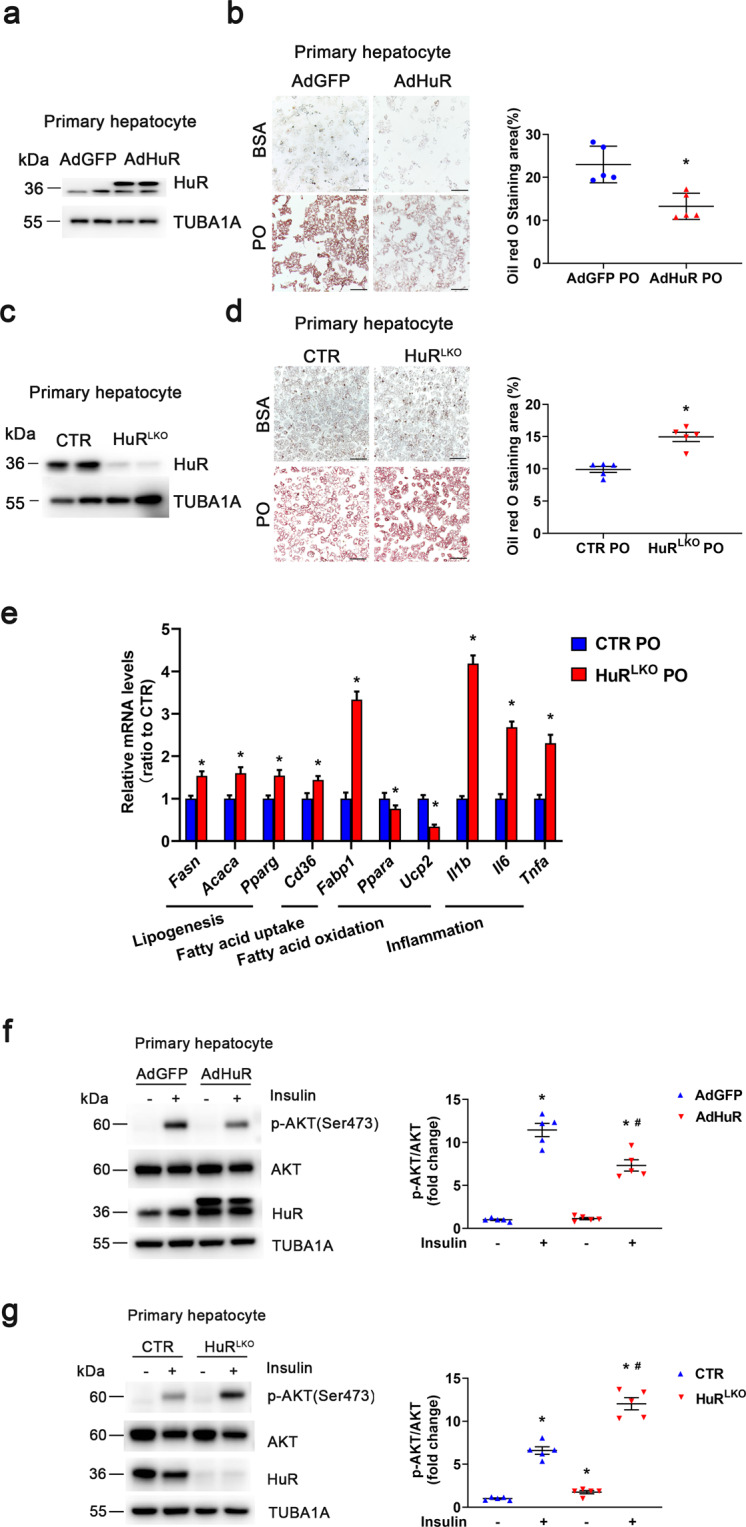
HuR inhibited lipid accumulation in hepatocytes. **a** Western blot analysis of HuR protein expression of primary hepatocytes infected with adenovirus expressing GFP or HuR for 24 h. **b** After infection with adenovirus for 24 h, primary hepatocytes were treated with BSA or PO for 24 h, followed by Oil-red O staining (*n* = 5). Scale bar, 50 μm. *P < 0.05 vs AdGFP PO. **c** Western blot analysis of HuR protein levels in primary hepatocytes from HuR^LKO^ and CTR mice. **d** Primary hepatocytes were treated with BSA or PO for 24 h, followed by Oil-red O staining (*n* = 5). Scale bar, 100 μm. *P < 0.05 vs CTR PO. **e** Relative mRNA expression of the indicated molecules in primary hepatocytes treated with PO (*n* = 5). *P < 0.05 vs CTR PO. **f** Western blot analysis of AKT phosphorylation in primary hepatocytes (from CTR mice) infected with AdGFP or AdHuR; the hepatocytes were then stimulated with insulin (10 µM) for 8 min (*n* = 5). *P < 0.05 vs AdGFP saline, ^#^*P* < 0.05 vs AdGFP insulin. **g** Western blot analysis of AKT phosphorylation in primary hepatocytes of CTR and HuR^LKO^ mice stimulated with insulin (10 µM) for 8 min (*n* = 5). *P < 0.05 vs CTR saline, ^#^*P* < 0.05 vs CTR insulin.

To determine the role of HuR in lipid accumulation in vitro, primary hepatocytes were infected with adenovirus expressing HuR followed by PO treatment. PO-induced lipid accumulation was attenuated by HuR overexpression (Fig. 4a, b). Consistently, primary hepatocytes from HuR-knockout mice showed aggravated PO-induced lipid accumulation compared to the controls (Fig. 4c, d). In primary hepatocytes stimulated with PO, HuR knockout significantly increased lipogenesis, fatty acid uptake, inflammation, and decreased fatty acid β-oxidation (Fig. 4e), which was consistent with the data from HuR^LKO^ mice fed the HFD. Consistent with the in vivo results, insulin-induced AKT phosphorylation in primary hepatocytes was suppressed by HuR overexpression (Fig. 4f) and enhanced with HuR deficiency (Fig. 4g). Thus, HuR regulates lipid metabolism in hepatocytes in vitro.

### HuR regulates *Pten* mRNA stability

**Fig. 5:**
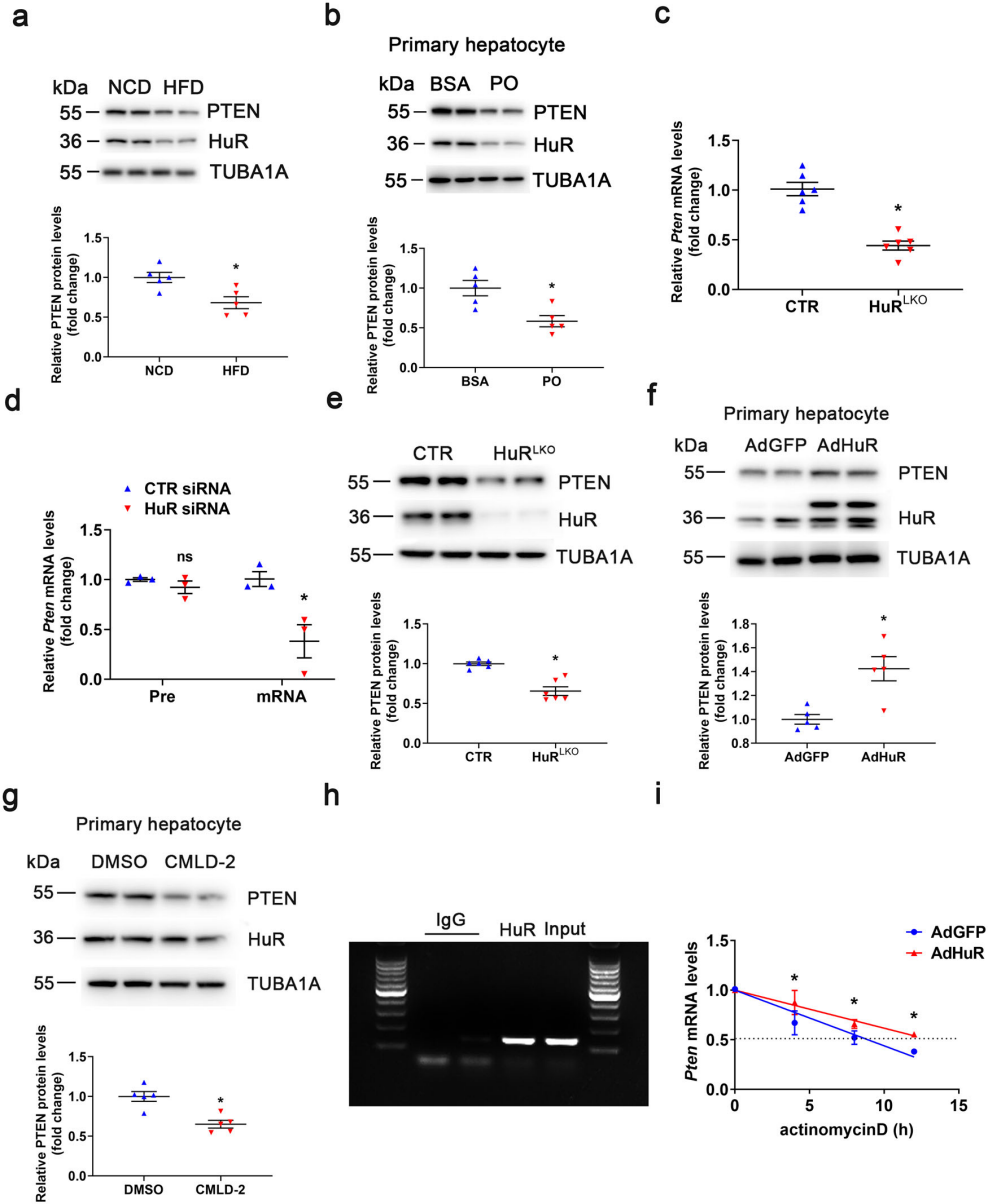
HuR regulates *Pten* mRNA stability. **a** Western blot analysis of PTEN protein levels in livers of NCD- or HFD-fed CTR and HuR^LKO^ mice (*n* = 5). *P < 0.05 vs NCD. **b** Western blot analysis of PTEN protein levels in primary hepatocytes stimulated with BSA or PO for 24 h (*n* = 5). *P < 0.05 PO vs BSA. **c** qPCR analysis to detect *Pten* mRNA levels in liver of CTR and HuR^LKO^ mice (*n* = 5). *P < 0.05 vs CTR. **d** qPCR analysis to detect *Pten* pre-mRNA and mature mRNA levels in primary hepatocytes transfected with CTR siRNA or HuR siRNA (*n* = 3). *P < 0.05 vs CTR siRNA. **e** Western blot analysis of PTEN protein levels in the liver of CTR and HuR^LKO^ mice (*n* = 5). *P < 0.05 vs CTR. **f** Western blot analysis of PTEN protein levels in primary hepatocytes infected with adenovirus expressing GFP or HuR for 24 h (*n* = 5). *P < 0.05 vs AdGFP. **g** Primary hepatocytes were stimulated with DMSO or CMLD-2 (30 μM) for 24 h. Western blot analysis of PTEN protein levels and quantification (*n* = 5). *P < 0.05 vs DMSO. **h** RNA immunoprecipitation with anti-HuR antibody or control IgG. **i** Primary hepatocytes infected with adenovirus expressing GFP or HuR for 24 h were stimulated with 10 μg/mL actinomycin d for the indicated time. qPCR analysis of *Pten* mRNA levels (*n* = 3). *P < 0.05 vs AdGFP.

HuR knockout in the liver impaired lipid metabolism and aggravated hepatic steatosis but improved insulin resistance in mice; the phenotype was similar to that observed in liver-specific *Pten* knockout mice^8,15^. Thus, we examined whether *Pten* is a target gene of HuR. The expression of PTEN was decreased in HFD mice and PO-stimulated primary hepatocytes (Fig. 5a, b), which was consistent with the HuR expression pattern. HuR deficiency reduced the level of mature *Pten* mRNA but did not affect its pre-mRNA level (Fig. 5c–e). In addition, PTEN protein levels were decreased by HuR knockout and increased by HuR overexpression (Fig. 5f). We examined the *Pten* mRNA sequence and identified 34 conserved AREs in the 3′ UTR of mouse *Pten* mRNA. The interaction between HuR and its target mRNAs could be disrupted by CMLD-2, which reduced PTEN protein levels (Fig. 5g). An RNA immunoprecipitation (RIP) assay demonstrated that HuR could bind to *Pten* mRNA (Fig. 5h). In addition, the half-life assay further confirmed that HuR overexpression increased *Pten* mRNA stability (Fig. 5i). Taken together, HuR binds to *Pten* mRNA and regulates its stability.

### HuR regulates hepatocyte steatosis through PTEN

**Fig. 6:**
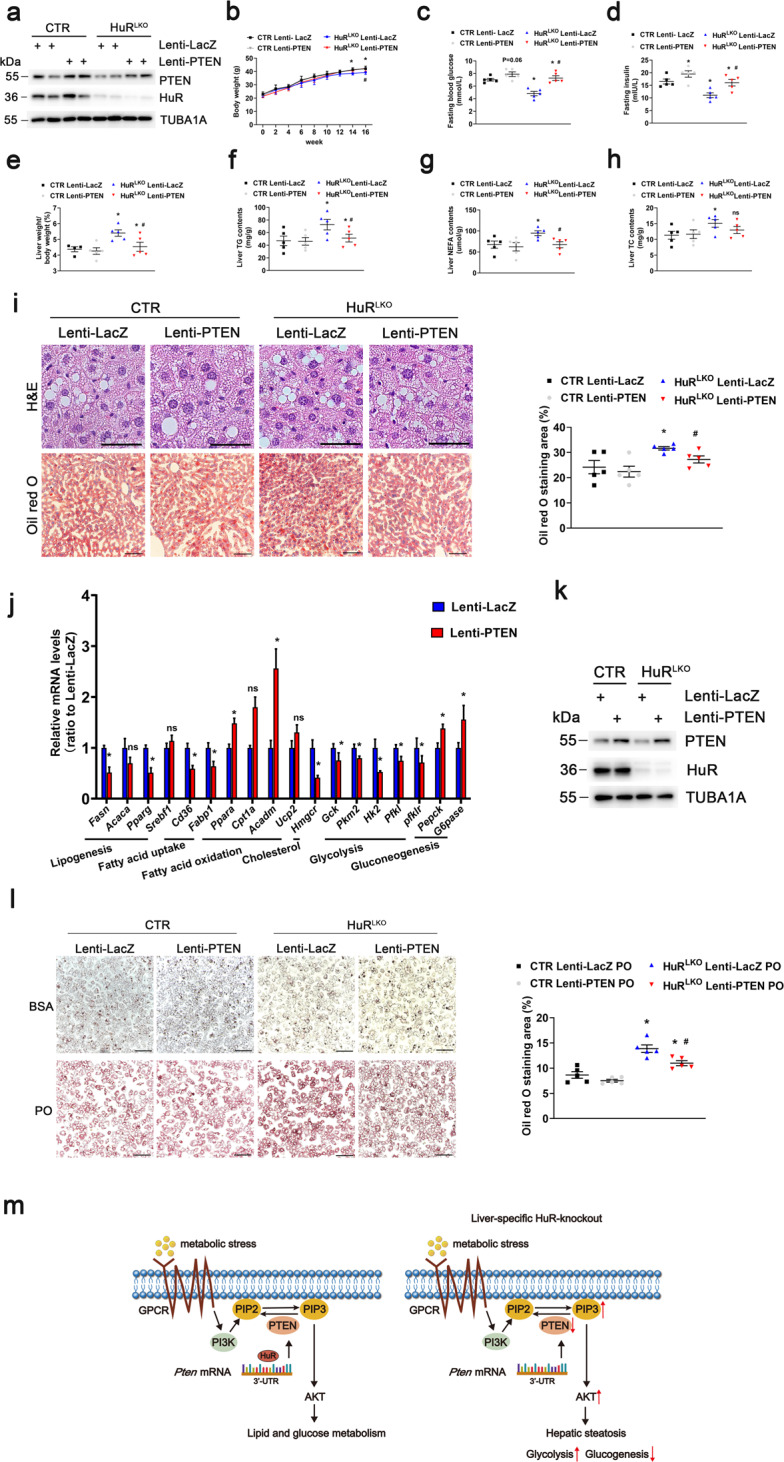
HuR regulates hepatocyte steatosis through PTEN. **a** HuR^LKO^ and CTR mice were injected with LacZ or PTEN lentivirus (1.0E + 07TU per mice) and fed a HFD. Western blot analysis of PTEN protein levels. **b** Body weight of mice in different groups (*n* = 5). *P < 0.05 vs CTR Lenti-LacZ, ^#^*P* < 0.05 vs HuR^LKO^ Lenti-LacZ. **c** Fasting glucose levels in the liver of mice (*n* = 5). *P < 0.05 vs CTR Lenti-LacZ, ^#^*P* < 0.05 vs HuR^LKO^ Lenti-LacZ. **d** Fasting insulin levels in the liver of mice (*n* = 5). *P < 0.05 vs CTR Lenti-LacZ, ^#^*P* < 0.05 vs HuR^LKO^ Lenti-LacZ. **e** Liver weight to body weight ratio of mice (*n* = 5). *P < 0.05 vs CTR Lenti-LacZ, ^#^*P* < 0.05 vs HuR^LKO^ Lenti-LacZ. TG (**f**), NEFA (**g**), and TC (**h**) levels in the liver of mice (*n* = 5). *P < 0.05 vs CTR Lenti**-**LacZ, ^#^*P* < 0.05 vs HuR^LKO^ Lenti-LacZ. **i** H&E-stained (top) and Oil-red O-stained (bottom) liver sections from mice. Scale bar, 50 μm. Quantitative analysis (right) of the mean Oil-red O-stained area (*n* = 5). *P < 0.05 vs CTR Lenti-LacZ, ^#^*P* < 0.05 vs HuR^LKO^ Lenti-LacZ. **j** Relative mRNA levels of the indicated molecules in the livers of mice (*n* = 5). *P < 0.05 vs HuR^LKO^ Lenti-LacZ. **k** Western blot analysis of PTEN protein levels. **l** CTR and HuR^LKO^ mice primary hepatocytes infected with PTEN lentivirus were treated with BSA or PO for 24 h, followed by Oil-red O staining (*n* = 3). Scale bar, 100 μm. *P < 0.05 vs CTR Lenti-LacZ PO, ^#^*P* < 0.05 vs HuR^LKO^ Lenti-LacZ PO. **m** Schematic diagram of the mechanism of HuR in lipid and glucose metabolism.

To examine whether HuR regulates hepatocyte steatosis through PTEN, control and HuR^LKO^ mice were injected with the lentivirus encoding LacZ (Lenti-LacZ) or PTEN (Lenti-PTEN) at week eight of the HFD. PTEN protein levels were significantly increased in the Lenti-PTEN groups (Fig. 6a). As expected, body weight and fasting blood glucose and insulin levels were significantly increased in HuR^LKO^ mice treated with Lenti-PTEN compared with Lenti-LacZ (Fig. 6b–d). Moreover, PTEN overexpression significantly decreased the LW/BW ratio, TG, NEFA, and TC levels in HuR^LKO^ mice (Fig. 6e–h). GTT and ITT indicated that HuR^LKO^ mice exhibited improved glucose tolerance and insulin resistance, which were exacerbated by PTEN overexpression (Supplementary Fig. 3a, b). However, the serum ALT and AST levels did not change after PTEN overexpression (Supplementary Fig. 3c). H&E and Oil Red O staining revealed a greatly decreased lipid deposition in the liver of HuR^LKO^ mice treated with Lenti-PTEN (Fig. 6i). In addition, Lenti-PTEN decreased the mRNA levels of genes associated with lipogenesis, FA uptake, cholesterol synthesis, and glycolysis, and increased the expression of genes related to FA β-oxidation and gluconeogenesis in HuR^LKO^ mice (Fig. 6j). PTEN overexpression significantly attenuated PO-induced lipid deposition in hepatocytes (Fig. 6l), further supporting the in vivo conclusion. In summary, hepatic HuR may modulate lipid and glucose metabolism by regulating PTEN expression.

## Discussion

In this study, we examined the effect of liver-specific HuR deletion on NAFLD. HuR^LKO^ mice were susceptible to the development of HFD-induced hepatic steatosis, but also displayed improved systemic insulin sensitivity. Mechanistically, HuR could bind to the 3′ UTR of *Pten* mRNA and increase its stability and translation. PTEN overexpression alleviated HFD-induced hepatic steatosis in HuR^LKO^ mice. Thus, HuR may play an important role in glycolipid metabolism by regulating PTEN expression.

HuR, an RNA-binding protein, mediates the expression of various proteins by modulating mRNA stability and translational efficiency^16^. Through its post-transcriptional effect on specific targets, HuR is involved in the cellular response to proliferation, stress, apoptosis, differentiation, senescence, as well as inflammatory and immune stimuli^17^. Recent studies have reported that HuR stabilizes cannabinoid receptor 1 and promotes cannabinoid receptor-mediated infiltration of bone marrow monocytes and macrophages in chronic liver injury^18^. In human hepatocellular carcinoma, elevated cytoplasmic HuR levels induced by TIP30 bind to *p53* mRNA 3′ UTR and induce apoptosis via stabilization of *p53* mRNA^19^. In this study, we generated liver-specific HuR knockout mice and found that HuR could protect against HFD-induced hepatic steatosis through PTEN, which broadens the biological functions of HuR.

The PTEN/AKT pathway plays a key role in the regulation of lipid and glucose metabolism. Therefore, it is important to explore the regulation of PTEN activity and expression. The catalytic activity of PTEN can be modulated post-translationally via acetylation and oxidation. Histone acetyltransferase and p300/CBP-associated factor-mediated acetylation results in the inactivation of PTEN activity^20^, which could also be caused by reactive oxygen species via oxidative stress-induced formation of a disulfide bond between Cys71 and Cys124^21,22^. In addition, different phosphorylation sites were regulated to control the activity of PTEN^23–25^. PTEN expression can be upregulated by many transcription factors, such as PPARG^26^ and early growth response protein 1^27^. In addition, numerous miRNAs including miR‑19 and miR‑21 could reduce PTEN levels in different diseases^28,29^. Here, we demonstrated that HuR, an mRNA-binding protein, could bind to the 3′ UTR of *Pten* mRNA and increase its stability and translation, which enhances our understanding of the regulatory mechanisms of PTEN expression.

Steatosis develops from increased de novo lipogenesis, FA uptake, and decreased very low-density lipoprotein export^8^. This process is closely linked to changes in hepatic glucose metabolism, including enhanced glycolysis (whose products are essentially used for de novo lipogenesis), as well as decreased gluconeogenesis. Alterations in a single organ can lead to marked phenotypic changes in the metabolic status of organisms via crosstalk between the liver and peripheral organs. Recently, another study to explore the role of HuR in NAFLD was published by Zhang and colleagues^14^. Consistently with our results, they reported that hepatic HuR deficiency aggravated HFD-induced NAFLD. However, we found that HuR expression was significantly decreased in liver of mice fed an HFD, which was opposite with Zhang’s study. The possible reason might be the different feeding condition of HFD. Besides, they demonstrated that HuR regulated lipid transport and ATP synthesis by associating with *Cycs*, *Ndufb6*, and *Uqcrb* mRNAs as well as *Apob* pre-mRNA, and thereby promoting the expression of CYCS, NDUFB6, UQCRB, and APOB. However, they did not explore hepatic steatosis related glucose metabolism. In our study, we found that liver-specific HuR deletion aggravated HFD-induced hepatic steatosis, but alleviated HFD-impaired glucose tolerance, which is similar to that found in liver-specific PTEN knockout mice^3,8,15^. In this context, the liver appeared to use more glucose for lipogenesis under enhanced insulin signaling; as a result, the liver stores more fat. PTEN-overexpressing mice were protected from steatosis^30,31^. As a positive regulator of PTEN, HuR is speculated to be a therapeutic target for NAFLD. Considering the opposing effects of HuR on lipid accumulation and insulin sensitivity, the combination of HuR overexpression with insulin sensitizers, such as thiazolidinediones, biguanides, glucagon-like peptide-1 receptor agonists, and dipeptidyl peptidase IV inhibitors, also known as incretins, might be a better therapeutic approach for the treatment of NAFLD^32,33^.

In summary, we demonstrated that HuR functions as an important regulator of lipid and glucose metabolism by targeting PTEN to increase its mRNA stability. Liver-specific HuR deletion aggravated HFD-induced hepatic steatosis, indicating that HuR may be a potential therapeutic target for NAFLD.

## Materials and methods

### Animals

C57BL/6J strain HuR^flox/flox^ (#021431) and albumin-Cre mice (#003574) were purchased from Jackson Laboratory (Bar Harbor, ME, USA). HuR^LKO^ mice were generated by crossbreeding HuR^flox/flox^ mice with albumin-Cre mice. Littermate HuR^flox/flox^/Cre^−^ mice were used as controls (CTR). When the mice were 8 weeks old, they were fed a HFD (TP23300; TROPHIC Animal Feed High-Tech Co. Ltd, China) consisting of 60% fat or a normal chow diet (NCD; TP23302; TROPHIC Animal Feed High-Tech Co. Ltd, China). Body weights were monitored weekly. All mice were housed under specific pathogen-free conditions on a 12-h light/12-h dark cycle with freely available food and water. Group allocation for the experiments was randomized and not blinded. Sample analyses were not blinded. Animal experiments were approved by the Animal Care Committee of Shandong University and were performed in compliance with the Animal Management Rules of the Chinese Ministry of Health. All procedures conformed to the guidelines of the NIH Guide for the Care and Use of Laboratory Animals.

### Reagents

Bovine serum albumin (BSA, #B2064), oleic acid (OA, #O1008), palmitic acid (PA, #P0500), MG-132(#M7449), CQ (#C6628), actinomycin D (#SBR00013), and the TG assay kit (MAK266) were purchased from Sigma-Aldrich (St. Louis, MO, USA). The HuR-ARE interaction inhibitor CMLD-2 was purchased from Millipore (#5.38339.0001). The antibodies used were specific to HuR (Millipore, # 07–468), AKT (Cell Signaling Technology, #4691), phospho-AKT at Ser473 (Cell Signaling Technology, #4060), PTEN (Cell Signaling Technology, #9188), TUBA1A (Proteintech, #11224-1-AP), and GAPDH (Proteintech, # 60004–1). Lentiviruses encoding PTEN (Lenti-PTEN) or Lenti-LacZ were purchased from Jikai (Shanghai, China). Adenovirus expressing green fluorescent protein (AdGFP) and HuR (AdHuR) were purchased from Vigenebio (MD, USA). The insulin ELISA kit was purchased from Mercodia (10-1249-01). The assay kits used to measure serum ALT, AST, NEFAs, and TC were purchased from Jiancheng Bioengineering Institute (Nanjing, China).

### Primary hepatocyte isolation and culture

Mouse primary hepatocytes were isolated from 8-week-old male CTR and HuR^LKO^ mice. In brief, after anesthetization, the liver was perfused through the portal vein with 40 ml warm (37°C) wash buffer (0.4 g/L KCl, 0.06 g/L KH_2_PO_4_, 8 g/L NaCl, 0.35 g/L NaHCO_3_, 0.132 g/L Na_2_HPO_4_.12H_2_O, 0.1% glucose, 1 mM Hepes, and 0.5 mM EDTA) at 5 ml/min with the perfusate exiting through the suprahepatic vena cava, followed by digestion with 20 ml collagenase type II buffer (Gibco, #17101-015). Hepatocytes were then placed in a 10-cm Petri dish. The cell suspension was isolated using a 100-μm filter (Falcon, #352360) and centrifuged at 800 rpm for 5 min. Hepatocytes were purified with 90% Percoll (GE Healthcare Life Sciences, #17-0891-01) and washed twice. Finally, hepatocytes were counted and cultured in high-glucose DMEM (Gibco, USA) containing 10% fetal bovine serum (FBS; BI, USA) at 37 °C in 5% CO_2_, followed by starvation for 6 h before treatment with fatty acid-free BSA or 0.5 mM PA along with 1.0 mM OA for a further 24 h before harvesting. Thereafter, 0.0307 g of PA was dissolved in 3 mL 0.1 M NaOH at 75 °C for 30 min to reach a concentration of 40 mM. The solutions were then mixed with 3 mL of 40% fatty acid-free BSA in phosphate-buffered saline (PBS) at 55 °C for 30 m in yielding a final stock solution of 20 mM. OA (19.04 μL) was dissolved in 3 mL NaOH (0.1 M) at 75 °C for 30 min to reach a concentration of 20 mM. The solutions were then mixed with 3 mL of 20% fatty acid-free BSA in phosphate-buffered saline (PBS) at 55 °C for 30 min, yielding a final stock solution of 10 mM. A control BSA solution was prepared by mixing NAOH with fatty acid-free BSA. All the stock solutions were stored at 4 °C.

### L02 cell culture

L02 cells, a line of normal human liver cells, were purchased from the Shanghai Institute of Cell Biology, Chinese Academy of Sciences (Shanghai, China) with STR authentication and cultured in RPMI-1640 medium containing 10% fetal bovine serum (FBS; BI, USA) at 37 °C in 5% CO2, followed by starvation for 6 h before treatment with fatty acid-free BSA or 0.5 mM PA along with 1.0 mM OA for a further 24 h before harvesting.

### GTT and ITT

For GTT, mice were fasted for 14–16 h and then administered an intraperitoneal (i.p.) injection of glucose (0.75 g/kg body weight). For ITT, mice were fasted for 4–6 h and then administered an i.p. injection of insulin (1.5 U/kg body weight). At 0, 15, 30, 60, 90, and 120 min after injection, blood glucose concentrations were measured.

### Liver histology

Livers were isolated, fixed in 4% formaldehyde solution, and embedded in paraffin wax. Then, 5-μm sections were cut and stained with H&E and periodic acid-Schiff (PAS). Immunohistochemical staining was performed using the HuR antibody (Millipore, # 07–468, 1:300). The fixed livers were dehydrated with sucrose and embedded in OCT. Frozen sections (10 μm) were stained with Oil Red O to assess hepatic steatosis.

### RNA immunoprecipitation assay

RIP assay was performed using a Magna RIP kit (Millipore, # 17–701). Cell lysates were treated with 5 μg rabbit IgG or HuR antibody and incubated with magnetic protein A/G beads at 4 °C overnight. The immunoprecipitated protein–RNA complex was washed and incubated with proteinase K buffer (30 min at 55 °C). RNA was extracted using phenol:chloroform:isoamyl alcohol, and reverse transcription was performed to synthesize cDNA. After PCR using the primers listed in Supplementary Table 1, the product was subjected to agarose gel electrophoresis.

### qPCR

TRIzol reagent (Invitrogen, Carlsbad, CA, USA) was used to extract total RNA from liver tissue or cells, according to the manufacturer’s protocol. The PrimeScript RT Reagent Kit (Takara Biomedical Technology) was used to reverse transcribe 1 μg of RNA into cDNA. PCR amplification was performed using SYBR PCR mix (Bio-Rad). The primer sequences used are listed in Supplementary Table 1.

### Western blot analysis

Briefly, tissue or cell lysates were run on 10% sodium dodecyl sulfate-polyacrylamide gel, and blots were probed with primary antibodies (1:1000) against HuR, AKT, phosphorylated AKT (Ser473), PTEN, TUBA1A, and GAPDH. Image J (National Institutes of Health, Bethesda, MD) was used to quantify the intensity of the bands.

### Statistical analysis

SPSS v23 (SPSS Inc., Chicago, IL, USA) was used for all analyses. All data are expressed as mean ± SEM and passed normality and equal variance tests. The comparison of two groups was determined using Student’s *t* tests and the comparison of multiple groups by one-way ANOVA with Bonferroni post-hoc tests. All statistical tests were two-tailed, and *P* < 0.05 was considered statistically significant.

## Supplementary information

Supplemental material
