# Supplementary material for: Hepatic HuR protects against the pathogenesis of non-alcoholic fatty liver disease by targeting PTEN
Source: Cell Death Dis. 2021 Mar 4;12(3):236. doi: 10.1038/s41419-021-03514-0 (PMC7933173; doi:10.1038/s41419-021-03514-0)
Supplement: Supplementary file 1 — Supplemental material [file 41419_2021_3514_MOESM1_ESM.docx]

**Hepatic HuR protects against the pathogenesis of non-alcoholic fatty liver disease** **by targeting PTEN**

Mi Tian^1,2^, Jingjing Wang^3^, Shangming Liu^4^, Xinyun Li^1^, Jingyuan Li^1^, Jianmin Yang^1^, Cheng Zhang^1^, Wencheng Zhang^1,2*^

^1^The Key Laboratory of Cardiovascular Remodeling and Function Research, Chinese Ministry of Education, Chinese National Health Commission and Chinese Academy of Medical Sciences, The State and Shandong Province Joint Key Laboratory of Translational Cardiovascular Medicine, Department of Cardiology, Qilu Hospital, Cheeloo College of Medicine, Shandong University, Jinan, China; ^2^Cardiovascular Disease Research Center of Shandong First Medical University，Central Hospital Affiliated to Shandong First Medical University; ^3^Department of Physiology & Pathophysiology, School of Basic Medical Sciences, Shandong University; ^4^Department of Histology and Embryology, School of Basic Medical Sciences, Shandong University

**Short Title:** Hepatic HuR modulates lipid and glucose metabolism

***Correspondence to:** Wencheng Zhang, No. 107, Wen Hua Xi Rd, Jinan, Shandong, China. 250012. Phone: 86-531-82169258. Fax: 86-531-82169257. E-mail: [zhangwencheng@sdu.edu.cn](mailto:zhangwencheng@sdu.edu.cn)

**Supplementary Figure 1.**


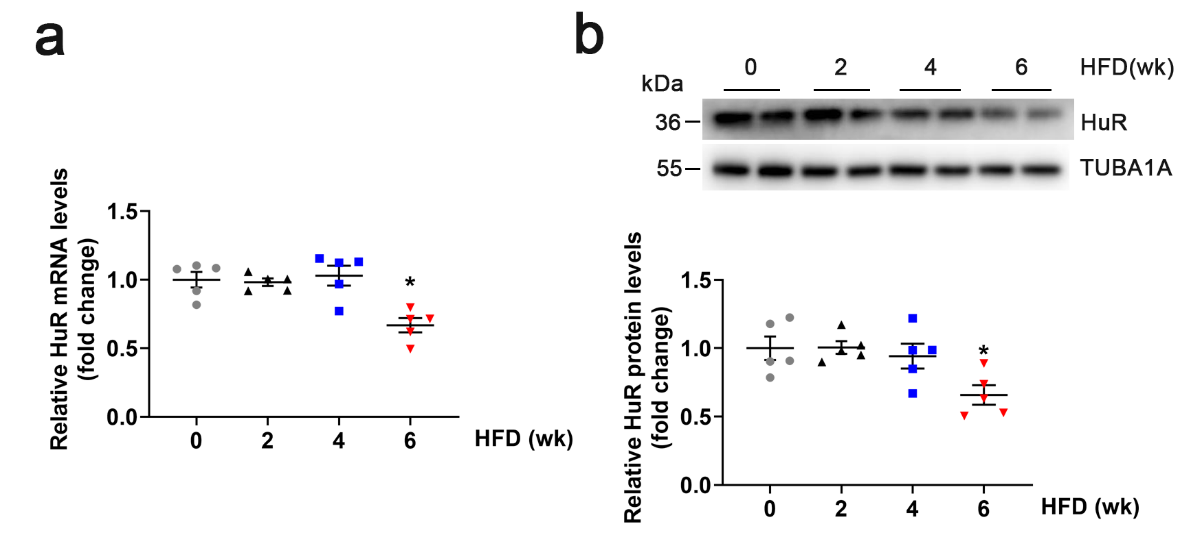


**Supplementary Figure 1.** **HuR mRNA and protein levels during the early stage of HFD feeding. (a)** qPCR analysis of HuR mRNA expression in livers of C57BL/6J mice fed a HFD for 0wk, 2wk, 4wk, or 6wk (n = 5). **P* <0.05 vs 0wk. **(b)** Western blot analysis of HuR protein levels in livers of C57BL/6J mice fed a HFD for 0wk, 2wk, 4wk, or 6wk (n = 5). **P* <0.05 vs 0wk.

**Supplementary Figure 2.**


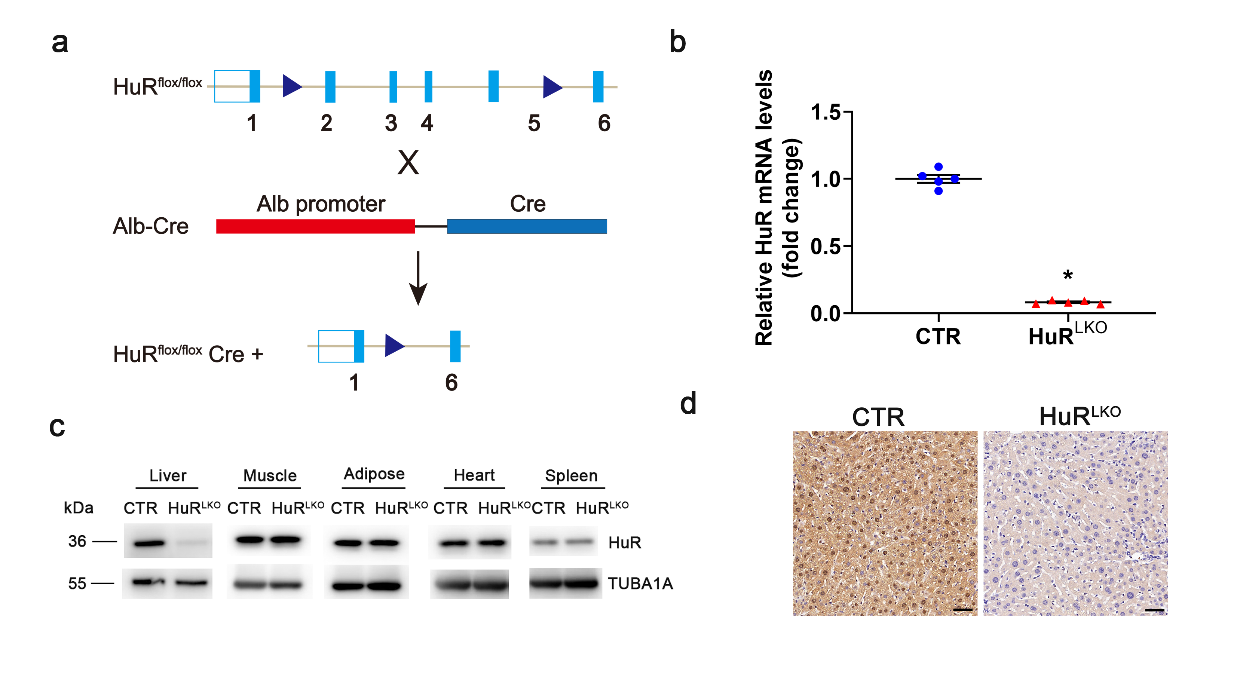


**Supplementary Figure 2. Preparation of liver-specific HuR-knockout mice (a)** Schematic diagram of transgenic mice used to generate HuR^LKO^ mice. **(b)** qPCR analysis of HuR mRNA levels in the liver of control and HuR^LKO^ mice (n = 5). **P* <0.05 vs CTR. **(c)** Western blot analysis of HuR protein levels in tissues from CTR and HuR^LKO^ mice. **(d)** Immunohistochemical staining of HuR protein in liver from CTR and HuR^LKO^ mice. Scale bar, 50 μm.

**Supplementary Figure 3.**


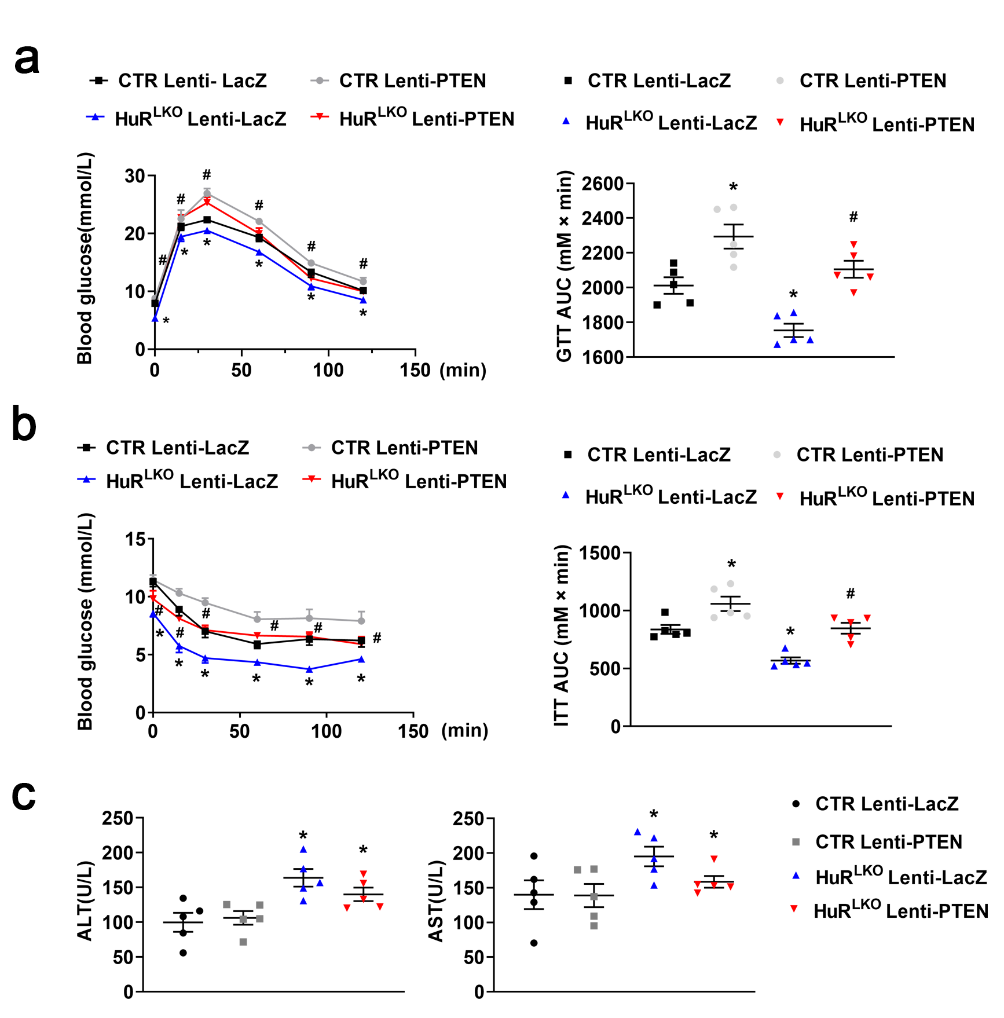


**Supplementary Figure 3. Improved glucose tolerance and insulin resistance in HuR^LKO^ mice were rescued by PTEN overexpression.** GTT **(a)** and ITT **(b)** in mice. The corresponding areas under the curve (AUC) of blood glucose levels (right) (n = 5). **P* <0.05 vs CTR Lenti-LacZ, ^#^*P* <0.05 *vs* HuR^LKO^ Lenti-LacZ. **(c)** Serum ALT and AST levels (n = 5). **P* <0.05 vs CTR Lenti-LacZ.

**Supplementary Table1. The primers for quantitative PCR.**

| Genes | Sequence 5’→3’ |
| --- | --- |
| *Pten*  *Hur*  *Fasn*  *Acaca* | F: GCAGCTTCTGCCATCTCTCTC  R: AACGATCTCTTTGATGATGGCTG  F: TGGGCGAATCATCAACTCCA  R: CGGATAAAGGCAACCCCTCT  F: CCAAGTGCAAGTGCAAACCA  R: GTTCCATGTTCACACGAGGC  F: GGGAACATCCCCACGCTAAA  R: GAAAGAGACCATTCCGCCCA |
| *Pparg*  *Srebf1* | F: ATTCTGGCCCACCAACTTCGG  R: TGGAAGCCTGATGCTTTATCCCCA  F: AGCAGTCACCAGCTTCAGTC  R: GGTCATGTTGGAAACCACGC |
| *Cd36* | F: GTCTATCTACGCTGTGTTCG |
| *Fabp1*  *Ppara*  *Cpt1a*  *Acadm*  *Ucp2* | R: ACAGGCTTTCCTTCTTTGC  F: AAAGTCAAGGCAGTCGTC  R: CCCAATGTCATGGTATTGGT  F: TATTCGGCTGAAGCTGGTGTAC  R: CTGGCATTTGTTCCGGTTCT  F: AAGAACATCGTGAGTGGCGT  R: ACCTTGACCATAGCCATCCAG  F: AAACATGGGCCAGCGATGCTCT  R: AGGGCATACTTCGTGGCTTCGT  F: CAATCTCGGGAGGCACCTTT  R: TGGGAAGTAAATCGGGGTGC |
| *Il1b* | F: AATGCCACCTTTTGACAGTGATG |
|  | R: ATGTGCTGCTGCGAGATTTG |
| *Il6* | F: AGTTGCCTTCTTGGGACTGA |
| *Tnfa*  *Hmgcr*  *Lxr* | R: TCCACGATTTCCCAGAGAAC  F: GTCCGGGCAGGTCTACTTTG  R: GGGGCTCTGAGGAGTAGACA  F: AGCTTGCCCGAATTGTATGTG  R: TCTGTTGTGAACCATGTGACTTC  F: ATTAAGGAAGAGGGGCAGGA  R: TGACCACGATGTAGGCAGAG |
| *Actb* | F: GTGACGTTGACATCCGTAAAGA |
| *Gck*  *Pkm2*  *Hk2*  *Pfkl*  *Pfklr*  *Pepck*  *Fbp1*  *Pcx*  *G6pase*  *Pten pre-mRNA*  *18s* | R: GCCGGACTCATCGTACTCC  F: GCGGAGATGCTCTTTGAC  R: GTCCCACGATGTTGTTCC  F: TCGCATGCAGCACCTGATT  R: CCTCGAATAGCTGCAAGTGGTA  F: TGATCGCCTGCTTATTCACGG  R AACCGCCTAGAAATCTCCAGA  F: CAGCTACGTGAAGGATCTG  R: CCATACCCATCTTGCTACTC  F: TGCACGACTCAACTTCTCCC  R: GGACTCCAGTGCGTATCTCG  F: CCACAGCTGCTGCAGAACA  R: GAAGGGTCGCATGGCAAA  F: AGTCGTCCTACGCTACCTGT  R: TGGTTCCGATGGACACAAGG  F: GCTATGAGGAGTTGGAAGAG  R: TGTTCCCATACTGGTCCC  F: CGACTCGCTATCTCCAAGTGA  R: GGGCGTTGTCCAAACAGAAT  F: GACAGCCATCATCAAAGAGATCG  R: AGAAATGCGCCCAGAATTAAACG  F: GGGAGCCTGAGAAACGGC  R: GGGAGTGGGTAATTT |
